# Sleep/wake estimation using only anterior tibialis electromyography data

**DOI:** 10.1186/1475-925X-11-26

**Published:** 2012-05-24

**Authors:** SuHwan Hwang, GihSung Chung, JeongSu Lee, JaeHyuk Shin, So-Jin Lee, Do-Un Jeong, KwangSuk Park

**Affiliations:** 1 Interdisciplinary program of Bioengineering, Seoul National University, Seoul, Republic of Korea; 2 Department of Psychiatry, Gyeongsang National University School of Medicine, Jinju, Republic of Korea; 3 Department of Neuropsychiatry and Center for Sleep and Chronobiology, Seoul National University Hospital, Seoul, Republic of Korea; 4 Department of Biomedical Engineering, College of Medicine, Seoul National University, Seoul, Republic of Korea

**Keywords:** Sleep/wake estimation, Electromyography, Sleep efficiency, Polysomonography

## Abstract

**Background:**

In sleep efficiency monitoring system, actigraphy is the simplest and most commonly used device. However, low specificity to wakefulness of actigraphy was revealed in previous studies. In this study, we assumed that sleep/wake estimation using actigraphy and electromyography (EMG) signals would show different patterns. Furthermore, each EMG pattern in two states (sleep, wake during sleep) was analysed. Finally, we proposed two types of method for the estimation of sleep/wake patterns using only EMG signals from anterior tibialis muscles and the results were compared with PSG data.

**Methods:**

Seven healthy subjects and five patients (2 obstructive sleep apnea, 3 periodic limb movement disorder) participated in this study. Night time polysomnography (PSG) recordings were conducted, and electrooculogram, EMG, electroencephalogram, electrocardiogram, and respiration data were collected. Time domain analysis and frequency domain analysis were applied to estimate the sleep/wake patterns. Each method was based on changes in amplitude or spectrum (total power) of anterior tibialis electromyography signals during the transition from the sleep state to the wake state. To obtain the results, leave-one-out-cross-validation technique was adopted.

**Results:**

Total sleep time of the each group was about 8 hours. For healthy subjects, the mean epoch-by-epoch results between time domain analysis and PSG data were 99%, 71%, 80% and 0.64 (sensitivity, specificity, accuracy and kappa value), respectively. For frequency domain analysis, the corresponding values were 99%, 73%, 81% and 0.67, respectively. Absolute and relative differences between sleep efficiency index from PSG and our methods were 0.8 and 0.8% (for frequency domain analysis). In patients with sleep-related disorder, our proposed methods revealed the substantial agreement (kappa > 0.61) for OSA patients and moderate or fair agreement for PLMD patients.

**Conclusions:**

The results of our proposed methods were comparable to those of PSG. The time and frequency domain analyses showed the similar sleep/wake estimation performance.

## Background

Polysomnography (PSG) has been regarded as the gold standard for sleep monitoring. In PSG recording, sleep stages and wakefulness are determined by electroencephalogram (EEG), electrooculogram (EOG) and chin electromyogram (EMG). PSG recording requires well-trained sleep experts, a controlled hospital environment, and a relatively long setup time, resulting in high costs. To overcome the disadvantages of PSG, in recent years, the need for home-based sleep monitoring has been specifically proposed [1-3]. In order to be successful, home-based sleep monitoring systems should be sensitive and sufficiently reliable to detect awake or sleep stages without depending on EEG.

**Figure 1:**
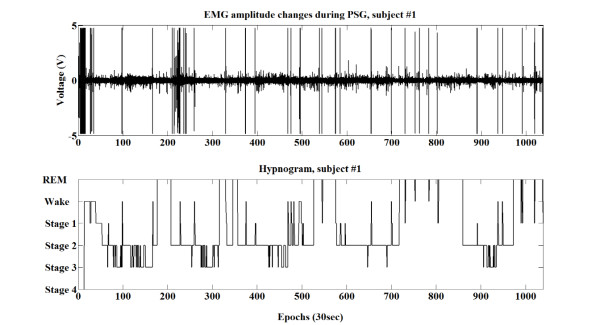
**EMG amplitude changes during PSG recording from subject #1.** The wake state is usually accompanied by a relatively high amplitude of EMG, as seen in the top figure. The bottom panel shows the hypnogram for comparison.

In general sleep monitoring systems, the results include sleep stages, sleep disorders, sleep duration, sleep fragmentation, sleep efficiency, sleep quality, etc. Among them, the continuous low sleep efficiency (numerous sleep disturbances) can lead to daytime fatigue [4], diabetes [5], headaches [6], and psychiatric disorders [7]. Therefore, it is important to precisely monitor sleep/wake patterns and sleep efficiency. In sleep efficiency measurement, actigraphy is the simplest device and has been used to estimate sleep/wake patterns in recent studies [8]. However, the validity of actigraphy to measure sleep efficiency is uncertain due to its limited ability to detect wakefulness [9]. The low specificity (around 50%) to wakefulness is basically caused by the assumption of the sleep state when subjects are simply inactive. Because of this drawback, other studies have proposed alternative ways to estimate the sleep/wake pattern without using an accele-ration sensor. These include the use of a heart rate measuring device [10], a mattress [11], a photographic monitoring device [12], mandible activity signals [13] and under-bed accelerometers [14]. In general, waking from sleep can be identified using physiological signal changes such as an abrupt increase in anterior tibialis EMG activities or heart rate [15,16]. The wake stage is usually accompanied by a relatively high tonic anterior tibialis EMG (shown in Figure 1). Also, a gradual diminution of the anterior tibialis EMG signal amplitude occurs during the transition from light to deep sleep [17]. However, use of anterior tibialis EMG signal changes to estimate sleep or wake patterns has rarely been studied.

In this paper, we assumed that sleep/wake estimation using actigraphy and EMG signals would show different patterns because the actigram is a collection of mechanical signals, whereas EMG signals are electrical. So we analysed EMG patterns in each state (sleep and wake during sleep).

This study was administered in two steps of sleep/wake pattern estimation. The first step was to establish methods for sleep/wake pattern estimation using only anterior tibialis EMG signals without EEG recordings. The second step was to confirm that the result of our methods is comparable to that of PSG.

## Methods

### Subjects and PSG data collection

Seven healthy subjects and five patients with sleep-related disorder participated in our study. Two patients (1 mild and 1 moderate) were diagnosed with obstructive sleep apnea (OSA) and the others were patients with periodic lime movement disorder (PLMD). A summary of the subject- and sleep- related parameters is shown in Table 1. Healthy subjects were free from any sleep-related disorders and it was verified by sleep physicians. All subjects reported no use of drugs or caffeine that could affect sleep and were given all the information about the purpose and methods of the study. All subjects have agreed that their PSG data, excluding personal information, will be used only for research purposes. PSG data were recorded and scored by registered polysomnographic technologists and physicians at Seoul National University Hospital according to the criteria described by Rechtschaffen and Kales [15]. A board certified sleep medicine physician made the final review of the PSG. The PSG data of all subjects were collected for about eight hours using the standard PSG routine and 15 channels of data. EEG electrodes were placed at the C3-A2 and O2-A1 positions based on the international 10–20 system. The following data were collected: EMG from the chin and bilateral tibialis anterior muscles, bilateral EOG, lead II electrocardiogram (ECG), nasal airflow, abdominal and thoracic movements, snoring, and blood oxygen saturation (SaO2). The PSG data were simultaneously acquired with the NI-DAQ 6221 (National Instruments, Austin, Texas) and sampling rate was 250 Hz. Based upon PSG data scoring, sleep stages were categorised into two criterions: wake and sleep. Stages 1, 2, 3, 4 and stage REM (rapid eye movement) were regarded as sleep.

**Table 1:** Summary of the sleep-related parameters of the subjects

**Parameter**	**Normal (mean ± SD)**	**Patients (mean ± SD)**
Sex (male/female)	4/3	4/1
Age (years)	33.3 ± 6.2	33.8 ± 13.2
BMI (kg/m^2^)	24.7 ± 5.4	24.2 ± 6.4
Sleep latency (min)	20.0 ± 22.8	16.0 ± 2.6
Stages 1 & 2 (%)	65.4 ± 7.9	66.6 ± 7.3
Stages 3 & 4 (%)	12.3 ± 8.1	10.0 ± 10.0
Stage REM (%)	22.3 ± 6.1	16.3 ± 5.7
Total sleep time (min)	454.7 ± 37.9	444.4 ± 41.4
Sleep efficiency (%)	97.6 ± 0.8	97.4 ± 1.9

*SD* standard deviation.

### Electromyography (EMG) signals

**Figure 2:**
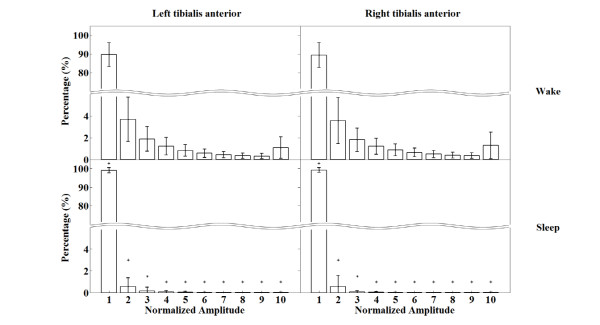
**Mean and standard deviation values of the normalised EMG amplitude histogram.** *Significant differences in normalised EMG amplitude between sleep and wake states (p < 0.05).

In the study, EMG signals from anterior tibialis muscles were chosen as indicators for our sleep/wake estimation methods because these signals reflect the awake movements. Therefore, EMG signals from each anterior tibialis muscle were used to estimate wakefulness using movement information. Differences in the sleep versus wake anterior tibialis EMG signal amplitude from subject #1 is shown in Figure 1. In accordance with the method of previous study [18], the EMG signal amplitudes during sleep and wake states were quantified after amplitude normalisation. As shown in Figure 2, the anterior tibialis EMG amplitude histogram shows significant differences between sleep and wake states: wake states show relatively high anterior tibialis EMG amplitudes compared with those in sleep states. Based on the anterior tibialis EMG amplitude differences between sleep and wake states, we were able to estimate sleep/wake cycles.

### Leave-one-out-cross-validation (LOOCV)

Leave-one-out-cross-validation method was used in this study. For the method, eleven EMG recordings were selected as a training set. Threshold selection factors (TSF) were computed based on training set data, and then threshold was applied to the last one (a testing set). This procedure was conducted twelve times (12 subjects participated in our study) and each result was obtained.

### Sleep/wake estimation methods: time domain analysis (TDA)

In TDA, the squared values of the anterior tibialis EMG signals from training sets were calculated, and all of the 250 samples of data were summed. In the following equation (1), x_j_ denotes the anterior tibialis EMG signals, y_k_ represents the summed data per second, at a sampling rate of 250 Hz. In Equation (2), z_l_ represents the sum of the y_k_ data per epoch (30 seconds).

(1) $$y_{k}=\sum_{j=(k-1)\times 250+1}^{k\times 250}x_{j}{}^{2}$$

(2) $$z_{l}=\sum_{k=(l-1)\times 30+1}^{l\times 30}y_{k}$$

From the training set EMG data, TSF_TDA1_ and TSF_TDA2_ which showed the best sleep/wake estimation performance in the training set data were calculated and applied to the testing set data. In subject #4 case (i.e. data from subject #4 was testing set), for example, TSF_TDA1_ and TSF_TDA2_ were 4 and 25, respectively. Consequently, the constant value 4 was multiplied by the average of z_l_ for 51 epochs (previous 25 epochs, current one epoch, subsequent 25 epochs) as a threshold T_m_ for the estimation (equation (3), m denotes the number of epoch). In this way, we were able to obtain the adaptive amplitude threshold of EMG signals for each epoch.

(3) $$\text{Threshold}\text{,}\;T_{m}=TSF_{\text{TDA}1}\times \frac{1}{\left(2\times TSF_{\text{TDA}2}+1\right)}(\sum_{l=(m-TSF_{\text{TDA}2})+1}^{l+TSF_{\text{TDA}2}}z_{l})$$

**Figure 3:**
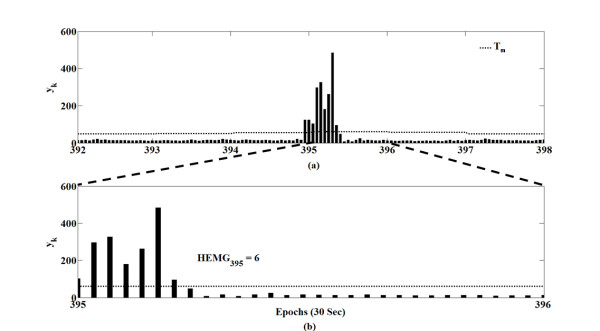
**An example of y**_**k**_**, threshold T**_**m **_**and HEMG**_**m **_**in subject #1.** (**a**) y_k_ signals from the 392^th^ epoch to the 397^th^ epoch; T_395_ was about 60. Bold lines indicate the y_k_ signals of each epoch, and the dashed line indicates the threshold T_m_. (**b**) Enlarged figure of epoch 395 in panel (a). In this case, HEMG_395_ was six.

**Figure 4:**
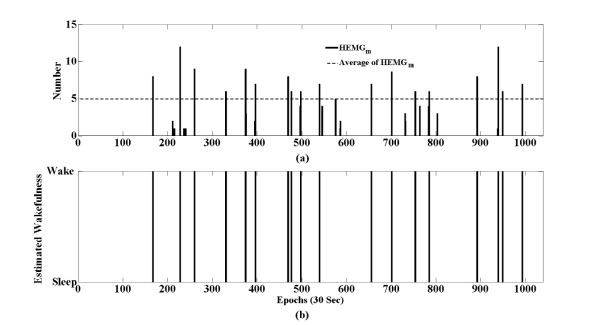
**HEMG**_**m **_**and the average HEMG**_**m **_**of the whole sleep period in subject #1.** (**a**) Bold lines indicate the HEMG_m_ of each epoch, and the dashed line indicates the average HEMG_m_. In the case of subject #1, the average HEMG_m_ was five. (**b**) Sleep/wake estimation using HEMG_m_ and the average of HEMG_m_. In the m^th^ epoch, if the HEMG_m_ exceeded the average of HEMG_m_, a wake state was concluded; otherwise, it was estimated as sleep.

The number of samples exceeding the threshold per epoch was calculated and named HEMG_m_. In equation (4), sgn(x) is 1 if x > 0; otherwise, it is 0. In the m^th^ epoch, if HEMG_m_ (the number of y_k_ over the threshold T_m_) exceeded the average of HEMG_m_, we estimated the m^th^ epoch as wake; otherwise, it was estimated as sleep. Figure 3 and Figure 4 show the data for y_k_, threshold T_m_, HEMG_m_ and the average HEMG_m_.

(4) $$\begin{array}{l} \text{The}\;\text{Number}\;\text{of}\;\text{High}\;\text{EMG}\;\text{Amplitude}\;\text{Sample}\text{,}\;\\ \;\text{HEM}G_{m}=\sum_{k=(m-1)\times 30+1}^{m\times 30}(\mathrm{sgn}(y_{k}-T_{m})) \end{array}$$

(5) $$\text{Average}\;\text{HEM}G_{m}=\frac{1}{N}(\sum_{m=1}^{N}\text{HEM}G_{m})\text{,}\;\text{N : total number of epochs}$$

### Sleep/wake estimation methods: Frequency domain analysis (FDA)

**Figure 5:**
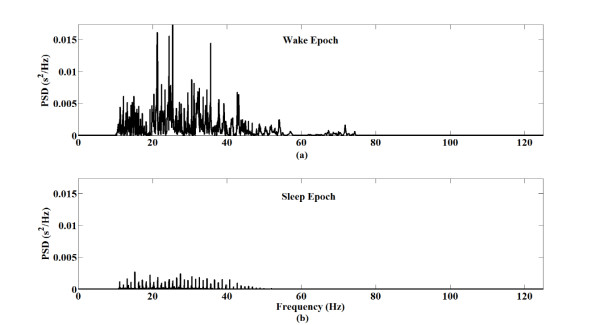
**Typical power spectrum density (PSD) of the EMG signals.** In this case, EMG data from the left tibialis muscles of subject #7 was used. PSD of each epoch were obtained using FFT and then the useless frequency powers (around 60 Hz, lower than 10 Hz, greater than 75 Hz) were ignored. (**a**) PSD of the EMG signals measured during wake epoch. (**b**) PSD of the EMG signals measured during sleep epoch.

In FDA, first, the total power of anterior tibialis EMG spectrum of each epoch from training sets was obtained using fast Fourier transform (FFT) and then the useless frequency powers (around 60 Hz, lower than 10 Hz, greater than 75 Hz) were ignored using MATLAB software (MathWorks, USA). Figure 5 shows a typical spectrum of the anterior tibialis EMG signals in subject #7. As shown in Figure 5 (a) and (b), the spectra of the anterior tibialis EMG signals measured during wake and sleep epochs are clearly different. Therefore, the total powers of the anterior tibialis EMG spectra of the sleep/wake epochs were easily differentiated.

(6) $$tp_{m}=\text{total}\;\text{power}\;\text{of}\;\text{the}\;\text{EMG}\;\text{spectrum}\;\text{of}\;\text{the}\;m^{\mathrm{th}}\;\text{epoch}$$

**Figure 6:**
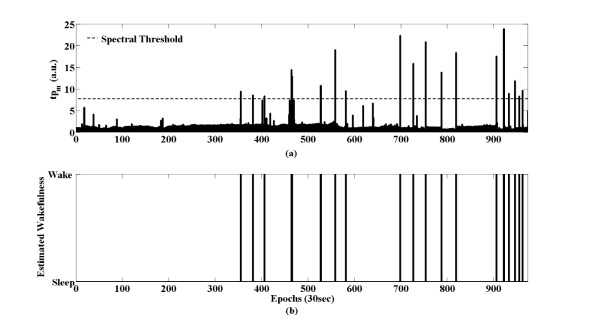
**Sleep/wake estimation with spectral threshold in subject #7.** (**a**) The total powers of the EMG spectrum of each epoch and the spectral threshold. (**b**) If t_pm_ exceeded the spectral threshold, the m^th^ epoch was estimated as wake; otherwise, it was estimated as sleep.

For sleep/wake estimation, tp_m_ (as defined in equation (6)) was compared with the spectral threshold. The spectral threshold was calculated as in equation (7). TSF_FDA1_ for average of the tp_m_ and TSF_FDA2_ for standard deviation of tp_m_ were decided from training set data. For instance, TSF_FDA1_ and TSF_FDA1_ were 0.5 and 3.5 in subject #1, respectively. We estimated the m^th^ epoch as wake if tp_m_ exceeded the spectral threshold and as sleep otherwise. The tp_m_ and spectral threshold are shown in Figure 6.

(7) $$\text{Spectral}\;\text{Threshold}=\text{TS}F_{\text{FDA}1}\times \text{mean}(tp_{m})+TSF_{\text{FDA}2}\times \text{std}(tp_{m})$$

### Statistical analysis

Epoch-by-epoch statistical analyses between PSG and the outcomes of our sleep/wake estimation algorithms were performed. For statistical analyses, seven statistical parameters (sensitivity [TP/(TP + FN)], specificity [TN/(FP + TN)], positive predictive value [TP/(TP + FP)], negative predictive value [TN/(TN + FN)], accuracy, kappa statistic and F-measure) were used. TP, TN, FP, and FN were further classified as ‘true positive,’ ‘true negative,’ ‘false positive,’ or ‘false negative,’ respectively. In our study, the sensitivity represents the proportion of correctly identified sleep states, while the specificity represents the proportion of correctly identified wake states. The kappa statistic value is most commonly used for studies that measure the agreement between two separate evaluators [19]. A kappa value in the range from 0.41 to 0.60 indicates moderate agreement, and one from 0.61 to 0.80 indicates substantial agreement. We also quantified the system performance using the F-measure, which considers both the precision and the recall of the test. Thus, this value indicates the harmonic mean of the sensitivity and specificity between two systems [20]. An F-measure score close to 1 signifies higher agreement between the two systems.

## Results

Our results from the two types of method for sleep/wake estimation were compared with PSG data. As shown in Table 2, the mean and standard deviations of the kappa value obtained via time and frequency domain analyses using EMG from bilateral tibialis muscles were 0.64 (0.06) and 0.67 (0.04) for normal subjects, respectively. For patient cases, corresponding values were 0.64 (0.00), 0.67 (0.01) in OSA and 0.39 (0.11), 0.46 (0.09) in PLMD. Six normal subjects (#1, 2, 3, 5, 6 and 7) and all OSA patients (#8 and 9) showed substantial agreement with regard to FDA. Six normal subjects (#1, 2, 3, 4, 5 and 6) and all OSA patients (#8 and 9) showed substantial agreement with regard to TDA. All other normal subjects demonstrated moderate agreement. For PLMD, subject #10 (in FDA) and 12 (in TDA) showed fair agreement.

**Table 2:** Statistical results of time and frequency domain analyses using EMG data from bilateral tibialis muscles

**Subject #(symptom)**	**Method**	**SENS**	**SPEC**	**PPV**	**NPV**	**ACCU**	**KAPPA**	**F-measure**
1	TDA	0.99	0.69	0.56	1.00	0.78	0.64	0.72
(N)	FDA	1.00	0.71	0.60	1.00	0.80	0.69	0.75
2	TDA	0.99	0.74	0.65	0.99	0.82	0.64	0.79
(N)	FDA	0.99	0.73	0.63	0.99	0.81	0.67	0.77
3	TDA	1.00	0.70	0.58	1.00	0.79	0.70	0.73
(N)	FDA	0.99	0.71	0.59	1.00	0.79	0.67	0.74
4	TDA	0.98	0.75	0.68	0.98	0.83	0.61	0.80
(N)	FDA	0.98	0.71	0.60	0.99	0.79	0.59	0.74
5	TDA	0.99	0.69	0.56	0.99	0.77	0.61	0.71
(N)	FDA	0.99	0.80	0.73	0.99	0.82	0.72	0.99
6	TDA	0.99	0.78	0.71	0.99	0.85	0.73	0.83
(N)	FDA	0.99	0.77	0.71	0.99	0.85	0.67	0.82
7	TDA	0.99	0.65	0.47	1.00	0.73	0.55	0.63
(N)	FDA	1.00	0.70	0.57	1.00	0.78	0.66	0.73
8	TDA	0.99	0.71	0.59	0.99	0.79	0.64	0.74
(O)	FDA	0.99	0.77	0.71	0.99	0.85	0.68	0.82
9	TDA	1.00	0.68	0.53	1.00	0.76	0.64	0.69
(O)	FDA	0.99	0.75	0.67	1.00	0.83	0.66	0.80
10	TDA	0.95	0.63	0.44	0.98	0.71	0.47	0.60
(P)	FDA	0.94	0.70	0.58	0.96	0.77	0.40	0.72
11	TDA	0.99	0.59	0.31	1.00	0.66	0.44	0.48
(P)	FDA	0.99	0.67	0.52	0.99	0.76	0.56	0.68
12	TDA	0.96	0.55	0.19	0.99	0.59	0.26	0.32
(P)	FDA	0.98	0.60	0.34	0.99	0.67	0.41	0.51
Mean(SD)	TDA	0.99 (0.01)	0.71 (0.04)	0.60 (0.08)	0.99 (0.01)	0.80 (0.04)	0.64 (0.06)	0.74 (0.07)
(N)	FDA	0.99 (0.01)	0.73 (0.04)	0.63 (0.06)	0.99 (0.01)	0.81 (0.02)	0.67 (0.04)	0.79 (0.09)
Mean(SD)	TDA	0.99 (0.01)	0.71 (0.04)	0.59 (0.08)	0.99 (0.01)	0.79 (0.04)	0.64 (0.05)	0.74 (0.06)
(N + O)	FDA	0.99 (0.01)	0.74 (0.04)	0.65 (0.06)	0.99 (0.01)	0.81 (0.03)	0.67 (0.03)	0.80 (0.08)
Mean(SD)	TDA	0.99 (0.02)	0.68 (0.07)	0.52 (0.15)	0.99 (0.01)	0.76 (0.07)	0.58 (0.13)	0.67 (0.15)
(N + O + P)	FDA	0.99 (0.02)	0.72 (0.05)	0.60 (0.11)	0.99 (0.01)	0.79 (0.05)	0.62 (0.11)	0.76 (0.11)

*TDA* time domain analysis, *FDA* frequency domain analysis, *SENS* sensitivity, *SPEC* specificity, *PPV* (positive predictive value, *NPV* negative predictive value, *ACCU* accuracy, *KAPPA* kappa statistic, *SD* standard deviation, symptom: *N* Normal, *O* OSA, *P* PLMD.

**Figure 7:**
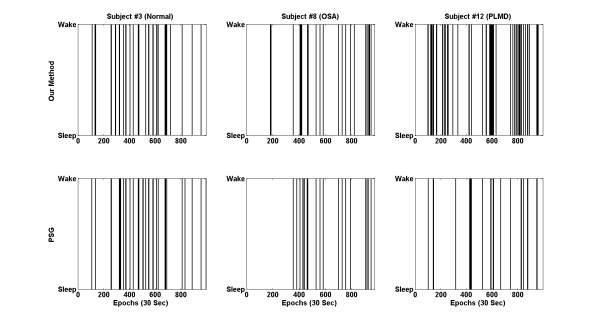
**Sleep/wake estimation results from subject #3, 8 and 12.** Sleep/wake patterns from the PSG recording are shown for reference. For these results, TDA method was used.

Figure 7 shows the sleep/wake estimation results from subject #3 (normal), 8 (OSA) and 12 (PLMD). The top figures indicate estimated sleep/wake patterns in TDA and the bottom figures show reference sleep/wake patterns from PSG. For PLMD patient, our proposed methods tended to overestimate the wake state.

Sleep/wake estimation results based on sleep postures are shown in Table 3. As shown in Table 3, there were no significant differences in sleep postures except prone posture (no one slept in prone posture). Kappa statistics showed similar value in the supine, right-lateral and left-lateral postures. In this analysis, TDA method was used.

**Table 3:** Sleep/wake estimation results based on sleep postures

**Subject # (symptom)**	**Supine**		**Lateral (left)**		**Lateral (right)**		**Prone**	
	**epoch**	**Kappa**	**epoch**	**Kappa**	**epoch**	**Kappa**	**epoch**	**Kappa**
1 (N)	627	0.68	173	0.60	177	0.52	0	n/a
2 (N)	756	0.63	0	n/a	81	0.65	0	n/a
3 (N)	681	0.72	137	0.69	122	0.61	0	n/a
4 (N)	756	0.60	0	n/a	81	0.65	0	n/a
5 (N)	687	0.56	277	0.70	0	n/a	0	n/a
6 (N)	959	0.71	0	n/a	44	0.76	0	n/a
7 (N)	849	0.55	0	n/a	0	n/a	0	n/a
8 (O)	592	0.63	0	n/a	383	0.64	0	n/a
9 (O)	863	0.66	0	n/a	110	0.61	0	n/a
10 (P)	641	0.42	155	0.51	151	0.46	0	n/a
11 (P)	565	0.56	336	0.38	0	n/a	0	n/a
12 (P)	900	0.25	79	0.46	0	n/a	0	n/a
Mean	739.6	0.58	96.4	0.56	95.8	0.61	0	n/a
(SD)	(128.8)	(0.13)	(119.5)	(0.13)	(109.9)	(0.09)	(0)	

*KAPPA* kappa statistic, *SD* standard deviation, symptom: *N* Normal, *O* OSA, *P* PLMD.

Sleep efficiency indexes from our methods were compared with the ones from PSG. Absolute and relative differences between each sleep efficiency index were calculated and the results are shown in Table 4. In this table, FDA method was applied and the mean values of relative differences were 0.8%. Especially, there were no difference between PSG and FDA for subject #4. For subject #10, the relative difference was 2.7% and it was the biggest difference in the whole case.

**Table 4:** Sleep efficiency index from PSG and our methods

**Subject #** **(symptom)**	**PSG S.E. (%)**	**Estimated S.E. (%)**	**Absolute difference**	**Relative difference (%)**
1 (N)	98.2	97.5	0.7	0.7
2 (N)	97.3	96.9	0.4	0.4
3 (N)	97.8	97.0	0.9	0.9
4 (N)	96.3	96.3	0.0	0.0
5 (N)	97.2	98.4	1.2	1.3
6 (N)	97.4	97.6	0.2	0.2
7 (N)	98.9	98.5	0.4	0.4
8 (O)	98.1	98.2	0.1	0.1
9 (O)	98.8	97.8	0.9	1.0
10 (P)	94.1	96.6	2.5	2.7
11 (P)	97.9	97.5	0.5	0.5
12 (P)	98.2	97.0	1.2	1.2
Mean(SD)	97.5 (1.3)	97.4 (0.7)	0.8 (0.7)	0.8 (0.7)

*S.E.* sleep efficiency, *SD* standard deviation, symptom: *N* Normal, *O* OSA, *P* PLMD.

Results from proposed methods in this study were compared with other sleep/wake estimation methods cited in the introduction section. In Table 5, results from heart rate-based method had similar performance as compared with our methods. In other cases (actigraphy, mandible activity, under-bed accelerometers), sensitivity and specificity from other methods were lower than those from our proposed methods. However, actigraphy- and under-bed accelerometers- based methods showed higher accuracy than our systems. In actigraphy-, heart rate- and under bed accelerometers- based studies, all participants were normal sleepers [9,10,14]. In mandible activity based method, all subjects were patients with sleep disorders [13].

**Table 5:** Overall sleep/wake estimation results from previous studies and ours

**Method**	**Actigraphy **[9]	**Heart rate **[10]	**Mandible activity **[13]	**Under-bed accelerometers **[14]	**Our method**
Number of participants	15	15	124	10	12
(N/S)	(15/0)	(15/0)	(0/124)	(10/0)	(7/5)
Sensitivity (%)	95.3	99.9	85.3	n/a	98.6
Specificity (%)	54.3	76.8	65.5	64.4	71.8
Accuracy (%)	90.7	n/a	n/a	95.2	79.3
Kappa	n/a	n/a	n/a	0.61	0.62

Number of participants: *N* Normal, *S* Sleep disorders.

## Discussion

### The agreement between our system and PSG

In this paper, we used anterior tibialis EMG signals to estimate the sleep/wake cycle and proposed two types of sleep/wake estimation methods using anterior tibialis EMG only: time domain analysis (TDA) and frequency domain analysis (FDA). Both TDA and FDA were applied and tested, and each estimation method showed high concordance with PSG. When EMG signals from bilateral tibialis muscle were used for sleep/wake cycle estimation, the mean kappa values of both methods revealed substantial agreement (above 0.61) for normal subjects and OSA patients. With both methods and in all subjects, kappa statistics showed greater than moderate agreement (above 0.41) except patient #10 and 12. Consequently, our sleep/wake estimation methods for normal subject and OSA patients proved to be comparable to PSG. Furthermore, the threshold was set according to subject anterior tibialis EMG tone and variance; therefore, the individual adaptive threshold could be applied to each subject and to each epoch. Because the proposed algorithms are simple and do not require trained sleep experts, if there exists with tibialis EMG recording, this approach can be used for portable or long-term (via a data storage device) sleep/wake pattern monitoring purpose. Moreover, these methods can support wake event detection during sleep in PSG recording.

### Sensitivity, specificity and kappa value

**Figure 8:**
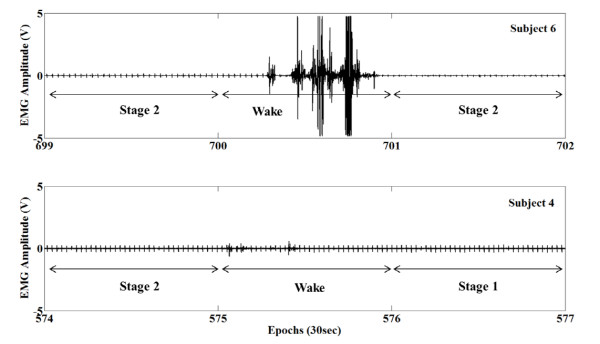
**EMG amplitude changes in sleep and wake states.** The top figure indicates EMG of subject #6 (699 to 702 epochs). The bottom figure indicates EMG of subject #4 (574 to 577 epochs). In the case, EMG signals from the left tibialis anterior muscles were used.

As shown in Tables 2, the specificity was lower than the sensitivity. Generally, in normal healthy subjects, the percentage of wake epochs is markedly less than the percentage of sleep epochs [17]. When we estimated the wake epochs, the specificity values were considerably reduced despite only a few wrong estimations. This may be due to the relative scarcity of wake epochs (see Table 1). So, in a non-symmetrical case of sleep/wake events, the accuracy cannot be used as an objective indicator [19]; therefore, we used kappa statistics to evaluate the performance of the sleep/wake estimation methods. Also, there were differences in sleep/wake estimation performance according to the subject. Figure 8 shows anterior tibialis EMG amplitude changes during the transition from sleep to wake. As shown, the anterior tibialis EMG amplitude changes of subject #4 were relatively small compared with those of subject #6. Therefore, in subject #4, the sleep/wake estimation performance was lower than that in subject #6 because our sleep/wake estimation methods were based on amplitude and frequency changes in anterior tibialis EMG signals. In this study, results were excellent when subjects were relatively active in the wake state and stayed immobile in the sleep state.

### Sleep efficiency index and sleep posture

In addition to the epoch-by-epoch analyses between PSG and our results, sleep efficiency indexes from our methods were calculated and these were compared with the ones from PSG (Table 4). Average of relative differences between each index was 0.8% and average absolute differences showed similar results. These results revealed that our systems are not only good estimator for the sleep/wake patterns but also useful estimator for the sleep efficiency index. Furthermore, in this study, we analysed the effects of sleep postures that could have influence on our sleep/wake estimation results (Table 3). However, there were no significant differences depending on sleep postures because the EMG electrodes were attached to the subject’s tibialis anterior muscles. Also, prone posture did not appear for all subject’s case. In PSG recording, a large number of electrodes are attached to the subject’s body (especially in the face) and this is why subjects can’t take the prone posture during sleep. So, in this analysis, sleep/wake estimation results in the prone posture could not be obtained.

### Patients with sleep-related disorder

Our proposed methods were applied to data of patients with sleep disorders. The results from OSA patients (1 mild severity and 1 moderate severity) showed similar results from healthy subjects (sensitivity: 0.99 ± 0.00, specificity: 0.76 ± 0.01, kappa: 0.67 ± 0.01, in FDA) because sleep/wake EMG patterns from OSA patients were no different from normal subjects. However, the sleep/wake estimation results from PLMD patients were lower than those from healthy groups (sensitivity: 0.97 ± 0.03, specificity: 0.66 ± 0.05, kappa: 0.46 ± 0.09, in FDA). For PLMD patients, our proposed methods tended to overestimate the wake state because it was based on the EMG tone and variance. In this case, the results showed moderate or fair agreement. PLMD is generally evaluated by measuring the EMG at the tibialis muscle [17]. So, for PLMD, our proposed systems may be more suitable for whole night anterior tibialis EMG signal tracking to detect PLM instead of sleep/wake pattern estimation. In previous studies, actigraphy signal or mandible activity based sleep/wake estimation methods had some ineffectiveness in the patients with PLMD or sleep-related breathing disorders [8,13,21].

### Anterior tibialis EMG signals and REM sleep

In normal adult humans, REM sleep is identified by a dearth of activity in the chin muscles, periodic bursts of rapid eye movement and the simultaneous existence of a relatively low-voltage cortical EEG [17]. Although anterior tibialis EMG signals are used to assess certain sleep-related disorders in general PSG, anterior tibialis EMG tone changes during REM sleep have not been established. In this study, tonic suppression of EMG signals from muscles beneath the chin was revealed in the transition from NREM sleep to REM sleep, but specific changes of anterior tibialis EMG tone were not occurred. So, to score macro sleep architecture (NREM, REM and wake) without EEG recordings, chin EMG signals are considered compulsory.

### Isometric muscular activity

Anterior tibialis EMG signal bursts are created by body movement; also, bursting anterior tibialis EMG signals are observed when subjects in the wake state lie immobile in bed, an occurrence known as ‘isometric muscular activity’ [22,23]. Therefore, anterior tibialis EMG signals might provide more data than actigraphy for inactive subjects in the wake state because actigraphy estimates sleep/wake patterns based on mechanical signals [9,24]. Consequently, our proposed methods showed better sleep/wake estimation performance than did actigraphy in previous studies with regard to specificity (actigraphy: around 50%, our system: around 70%) and showed high concordance with PSG [9,25].

### Drawbacks

Despite their advantages, our methods also have limitations. For patients with periodic limb movement in sleep (PLMS), our methods tend to estimate the sleep state as wake because they are anterior tibialis EMG tone and variance based and are influenced by body movement. Our proposed system may not be as user-friendly as actigraphy because EMG electrodes must be attached to the tibialis anterior muscles. To overcome these drawbacks, non-intrusive sensors for PLM detection and physiological signal monitoring [26,27] can be applied in our system.

## Conclusions

Our proposed methods show that it is possible to estimate sleep/wake states using only anterior tibialis EMG data. Using the surface EMG electrodes, we were able to obtain a sleep/wake estimation performance comparable to that of PSG. We plan to focus our future study on non-intrusive sleep/wake estimation using EMG in order to relieve the inconvenience of electrode attachment.

## Competing interests

The authors declare that they have no competing interests.

## Authors’ contributions

SHH developed the methods of this study and drafted the manuscript. GSC and JSL established the data acquisition system. JHS and SJL helped analysis and interpretation of the results. DUJ and KSP reviewed the manuscript as corresponding author. All authors read and approved the final manuscript.
